# Profile of Acute Kidney Injury Patients Admitted to a Tertiary Care Centre in Urban South India: A Prospective Observational Study

**DOI:** 10.7759/cureus.111253

**Published:** 2026-06-21

**Authors:** Joby Wilson, Seetha Rami Reddy Mallampati, Varunkrishnan G, Ganesan C

**Affiliations:** 1 General Medicine, Vinayaka Missions Medical College and Hospital, Vinayaka Mission’s Research Foundation (Deemed to be University), Karaikal, IND

**Keywords:** acute kidney injury, hospital mortality, kdigo criteria, renal recovery, sepsis

## Abstract

Background: Acute kidney injury (AKI) is a common clinical syndrome associated with significant morbidity, mortality, and healthcare burden. Data regarding the prevalence, causes, and outcomes of AKI in tertiary care settings in South India remain limited. This study aimed to evaluate the prevalence, aetiology, clinical profile, and in-hospital outcomes of AKI among adults admitted to a tertiary care centre.

Methods: This prospective observational study was conducted over 18 months among adult patients admitted to the general medicine wards of a tertiary care hospital in South India. Patients fulfilling the Kidney Disease: Improving Global Outcomes (KDIGO) 2012 criteria for AKI were enrolled. Demographic, clinical, laboratory, and outcome data were collected using a structured proforma. AKI was classified as pre-renal, intrinsic renal, or post-renal based on clinical and laboratory findings. Statistical analysis was performed using SPSS version 25.0 (IBM Corp., Armonk, NY, USA), with p<0.05 considered statistically significant.

Results: Among 2,427 hospital admissions, 65 patients met KDIGO criteria for AKI, yielding a prevalence of 2.67%. Most patients were male (44, 67.7%), and the most affected age group was 51-65 years (24, 36.9%). Type 2 diabetes mellitus (29, 44.6%) and hypertension (26, 40.0%) were the most common comorbidities. Sepsis was the leading aetiology (32, 49.2%), followed by cardiac failure (12, 18.5%) and chronic liver disease (5, 7.7%). Pre-renal AKI accounted for 34 (52.3%) cases, intrinsic renal AKI for 28 (43.1%), and post-renal AKI for three (4.6%). Complete renal recovery occurred in 28 (43.1%) patients, partial recovery in 22 (33.8%), and in-hospital mortality in 15 (23.1%). Oliguria (p=0.01), hypotension (p<0.001), elevated leukocyte count (p<0.01), blood urea nitrogen (p<0.01), peak serum creatinine (p<0.01), urine sodium (p=0.01), and fractional excretion of sodium (p<0.01) were significantly associated with adverse outcomes.

Conclusion: AKI remains an important cause of morbidity and mortality among hospitalised adults. Sepsis was the predominant aetiology, while hypotension, oliguria, and severe biochemical abnormalities were associated with poor outcomes. Early recognition and prompt management of high-risk patients may improve renal recovery and reduce mortality.

## Introduction

Acute kidney injury (AKI) is a clinical syndrome characterised by an abrupt decline in renal excretory function, resulting in retention of nitrogenous waste products, dysregulation of fluid and electrolyte homeostasis, and systemic organ dysfunction. The Kidney Disease: Improving Global Outcomes (KDIGO) criteria [1] define AKI as a rise in serum creatinine of ≥0.3 mg/dL within 48 hours, a ≥1.5-fold increase from baseline within seven days, or urine output below 0.5 mL/kg/h for six or more hours - criteria that have enabled standardised diagnosis and meaningful cross-study comparisons [2].

AKI represents a significant burden on healthcare systems globally. Prevalence estimates vary widely, from less than 1% to over 60% of hospitalised patients, depending on the diagnostic criteria applied, clinical setting, and patient population studied [3]. In intensive care units, AKI complicates the course of 50-60% of critically ill patients and is independently associated with prolonged hospital stay, increased need for renal replacement therapy (RRT), and high in-hospital mortality [4]. In developing countries, including India, community-acquired AKI predominates, with sepsis, hypovolaemia, and nephrotoxin exposure constituting the principal aetiologies [5].

Sepsis-associated AKI deserves particular attention, as sepsis accounts for nearly 50% of all AKI in critically ill patients [6]. The pathophysiological mechanisms underlying sepsis-AKI microvascular dysfunction, dysregulated inflammation, and metabolic reprogramming remain incompletely understood, limiting the development of targeted therapies [7]. Beyond the acute episode, AKI carries substantial long-term consequences; survivors face a significantly elevated risk of developing chronic kidney disease (CKD), end-stage renal disease, and all-cause mortality extending years beyond discharge [8].

Tertiary care hospitals, with their complex and high-acuity patient mix, represent an important epidemiological lens through which to characterise the local burden, aetiology spectrum, and outcomes of AKI. Understanding these patterns at the institutional level is essential for developing context-appropriate preventive strategies and optimising clinical management. This study therefore aims to evaluate the prevalence, aetiology, and outcomes of AKI in patients admitted to a tertiary care centre in South India.

## Materials and methods

This was a prospective observational study conducted in the inpatient wards of Vinayaka Mission's Medical College and Hospital (VMMCH), Karaikal, a tertiary care institution in Puducherry, India, over a period of 18 months (January 2024 to June 2025). The study was approved by the Institutional Ethics Committee prior to initiation (approval VMMC/2022/July/38), and written informed consent was obtained from all participants or their legally authorized representatives before enrollment. The study was conducted in accordance with the Declaration of Helsinki.

Adult patients aged 18 years and above who were admitted to the general medicine wards during the study period and who met the KDIGO 2012 criteria [1] for AKI were considered eligible for inclusion. AKI was defined as any of the following: a rise in serum creatinine of ≥0.3 mg/dL within 48 hours; an increase in serum creatinine to ≥1.5 times the baseline value within the preceding seven days; or a urine output of less than 0.5 mL/kg/h for six or more consecutive hours. Patients with pre-existing CKD (defined as a known history of renal impairment prior to the index admission or evidence of chronically elevated creatinine on medical records) and those with obstetric AKI were excluded, as these subgroups carry distinct pathophysiological mechanisms and management considerations that would confound the primary analysis. For patients without documented previous serum creatinine measurements, baseline renal function was assessed using available medical records, history of CKD, previous nephrology consultations, ultrasonographic evidence of chronic renal parenchymal disease (such as reduced kidney size or increased cortical echogenicity), and clinical evaluation. Patients with findings suggestive of underlying CKD were excluded from the study. Only patients in whom acute deterioration of renal function was considered clinically consistent with AKI according to KDIGO criteria were included.

During the study period, all consecutive adult patients admitted to the general medicine wards who fulfilled the KDIGO 2012 diagnostic criteria for AKI were screened for eligibility. Based on hospital admission statistics and the expected number of AKI cases presenting to the institution during the 18-month study period, it was anticipated that approximately 60-70 eligible patients would be available for enrollment. Therefore, all eligible patients meeting the inclusion criteria were recruited consecutively until completion of the study period. A total of 65 patients were enrolled and included in the final analysis. For each enrolled patient, a structured proforma was completed at the time of admission and updated serially throughout the hospital stay until discharge or death.

Demographic data collected included age, sex, and place of residence. A detailed medical history was taken, documenting comorbid conditions (including type 2 diabetes mellitus, systemic hypertension, coronary artery disease, chronic liver disease, and hypothyroidism), current medications, and any prior episodes of AKI. Presenting symptoms recorded at admission included oliguria, anuria, oedema, fever, vomiting, diarrhoea, hypotension, jaundice, haematuria, dyspnoea, trauma, rash, and history of nephrotoxic drug or poison exposure.

All patients underwent a standardised set of investigations at admission and serially during hospitalisation. These included a complete blood count (with total leucocyte count and platelet count), blood urea nitrogen, serum creatinine, serum sodium, serum potassium, urine routine and microscopy, spot urine sodium, and calculation of the fractional excretion of sodium (FENa), using the formula: FENa (%) = [(urine sodium × serum creatinine) / (serum sodium × urine creatinine)] × 100. Serum creatinine was measured daily from admission until discharge or death to track the trajectory of renal function over time. Additional investigations, including liver function tests, coagulation profile, blood and urine cultures, chest radiograph, 2D echocardiography, abdominal ultrasonography, arterial blood gas analysis, and renal biopsy, were performed selectively based on clinical indication, as recorded in the case proforma.

The aetiology of AKI was determined through integration of clinical findings, laboratory results, and imaging, and was classified into three categories: pre-renal (caused by states of reduced renal perfusion such as sepsis, cardiac failure, hypovolaemia, or hepatorenal syndrome), intrinsic renal (caused by direct parenchymal injury, including acute tubular necrosis, glomerulonephritis, and interstitial nephritis), and post-renal (caused by obstructive uropathy). Management during hospitalisation was recorded, including all fluid therapies, antibiotics, inotropic support, and any RRT. Where haemodialysis was instituted, the indication, modality, and frequency of sessions were documented.

Clinical outcomes were assessed at the point of hospital discharge or death. Outcomes were classified into three categories: full recovery (return of serum creatinine to within 0.3 mg/dL of the pre-admission baseline with adequate urine output), partial recovery (improvement in creatinine and urine output but failure to return to baseline), and death (in-hospital mortality). Patients were monitored continuously from admission to the outcome endpoint; there was no post-discharge follow-up in this study.

All data were entered into Microsoft Excel (Redmond, WA, USA) and subsequently analysed using SPSS version 25.0 (IBM Corp., Armonk, NY, USA). Continuous variables were tested for normality using the Shapiro-Wilk test and expressed as mean ± standard deviation. Differences in continuous variables across outcome groups were examined using one-way ANOVA or the Kruskal-Wallis H test, as appropriate. Categorical variables were expressed as frequencies and percentages, and between-group differences were assessed using the chi-square test. A two-tailed p-value of less than 0.05 was considered statistically significant for all analyses.

## Results

Of 2,427 adult admissions to the General Medicine wards during the study period, 65 patients met KDIGO criteria for AKI, corresponding to a prevalence of 2.67% among General Medicine admissions. Full renal recovery was achieved in 28 patients (43.1%), partial recovery in 22 (33.8%), and 15 patients (23.1%) died during hospitalisation. A total of 61 patients (93.8%) were managed conservatively; haemodialysis was required in four patients (6.2%), with no significant difference in its use across outcome groups (p=0.29). The demographic and clinical characteristics of the cohort are summarised in Table 1. Of 65 patients, 44 (67.7%) were male and 21 (32.3%) were female. The 51-65 year age group was most frequently affected, comprising 24 patients (36.9%), followed by the 66-80 year group (18, 27.7%). Type 2 diabetes mellitus was present in 29 patients (44.6%) and systemic hypertension in 26 patients (40.0%). Neither age distribution nor sex differed significantly across outcome groups (p=0.67 and p=0.74, respectively).

**Table 1:** Baseline demographic and clinical characteristics of study participants (n=65) Values are expressed as frequency (percentage). Comorbidity categories are not mutually exclusive.

Characteristic	n (%)
Age group (years)	
18-35	5 (7.7)
36-50	14 (21.5)
51-65	24 (36.9)
66-80	18 (27.7)
>80	4 (6.2)
Sex	
Male	44 (67.7)
Female	21 (32.3)
Comorbidities	
Type 2 diabetes mellitus	29 (44.6)
Systemic hypertension	26 (40.0)
Chronic liver disease	7 (10.8)
Others	6 (9.2)
Coronary artery disease	5 (7.7)
Hypothyroidism	3 (4.6)

The distribution of presenting symptoms by outcome is shown in Table 2. Oedema was recorded in 27 patients (41.5%), oliguria in 16 (24.6%), and hypotension in 15 (23.1%). Oliguria and hypotension were significantly associated with worse outcomes (p=0.01 and p<0.001, respectively); all other presenting symptoms did not reach statistical significance.

**Table 2:** Presenting symptoms and their association with clinical outcome (n=65) p-values derived from chi-square test. Categories are not mutually exclusive. "History of trauma" denotes a preceding traumatic event as a precipitating cause, not a presenting symptom per se.

Symptom	Full recovery (n=28) n (%)	Partial recovery (n=22) n (%)	Death (n=15) n (%)	Total (n=65) n (%)	p-value
Oedema	11 (39.3)	10 (45.5)	6 (40.0)	27 (41.5)	0.89
Oliguria	2 (7.1)	8 (36.4)	6 (40.0)	16 (24.6)	0.01
Hypotension	1 (3.6)	2 (9.1)	12 (80.0)	15 (23.1)	<0.001
Fever	11 (39.3)	5 (22.7)	3 (20.0)	19 (29.2)	0.29
Vomiting	6 (21.4)	4 (18.2)	1 (6.7)	11 (16.9)	0.46
History of trauma	8 (28.6)	2 (9.1)	1 (6.7)	11 (16.9)	0.07
Jaundice	3 (10.7)	3 (13.6)	3 (20.0)	9 (13.8)	0.70
Diarrhoea	3 (10.7)	3 (13.6)	1 (6.7)	7 (10.8)	0.79
Nephrotoxic drug exposure	4 (14.3)	1 (4.5)	1 (6.7)	6 (9.2)	0.64

**Figure 1:**
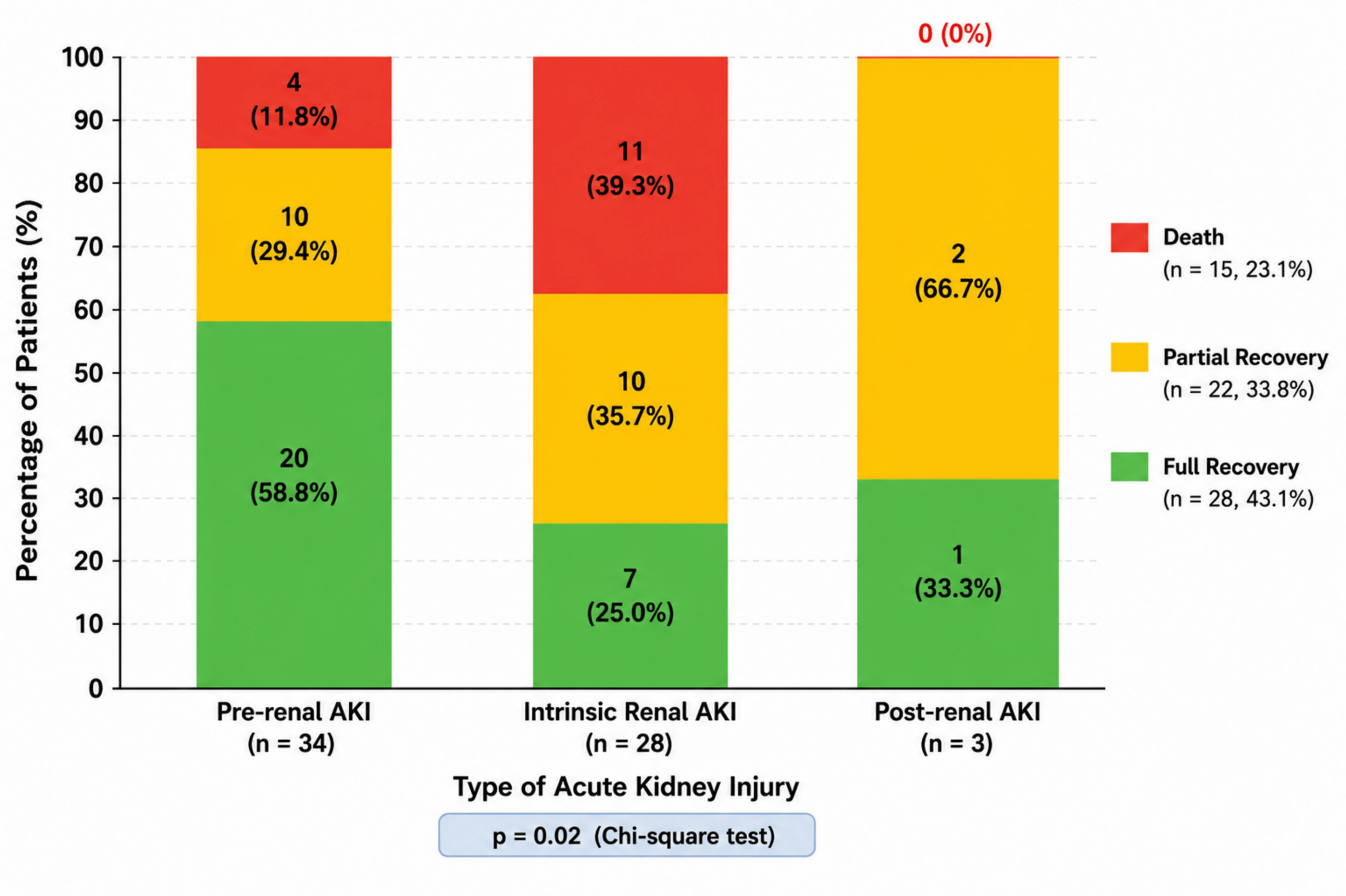
Distribution of clinical outcomes according to acute kidney injury subtype.

Sepsis was the underlying aetiology in 32 patients (49.2%), followed by cardiac failure in 12 (18.5%), chronic liver disease in five (7.7%), stroke in four (6.2%), acute gastroenteritis and post-renal obstruction in three patients each (4.6%), and miscellaneous causes including burns, carcinoma oesophagus, systemic lupus erythematosus, sarcoidosis, alcoholic hepatitis, and malignant hypertension in one patient each (1.5%). Pre-renal AKI was identified in 34 patients (52.3%), intrinsic renal AKI in 28 (43.1%), and post-renal AKI in three (4.6%). The distribution of renal failure type differed significantly across outcome groups (p=0.02); pre-renal AKI was present in 20 of 28 patients (71.4%) who achieved full recovery, whereas intrinsic renal AKI was present in 11 of 15 patients (73.3%) who died. The distribution of clinical outcomes according to AKI subtype is shown in Figure 1.

Laboratory parameters stratified by outcome are presented in Table 3. Total leucocyte count, blood urea nitrogen, peak serum creatinine, urine sodium, and FENa differed significantly across outcome groups (p<0.01, p<0.01, p<0.01, p=0.01, and p<0.01, respectively). Baseline serum creatinine approached but did not reach statistical significance (p=0.05). Platelet count, serum sodium, and serum potassium showed no significant differences across groups.

**Table 3:** Laboratory parameters by clinical outcome group (n=65) Values expressed as mean ± standard deviation. p-values derived from one-way ANOVA

Parameter	Full recovery (n=28) Mean ± SD	Partial recovery (n=22) Mean ± SD	Death (n=15) Mean ± SD	Total (n=65) Mean ± SD	p-value
Total leucocyte count (cells/μL)	14441.07 ± 8322.05	9868.97 ± 5021.48	23461.33 ± 18715.71	14975.19 ± 11835.56	<0.01
Platelet count (/μL)	232214.29 ± 113052.38	239531.82 ± 154411.55	221533.33 ± 177122.98	232226.15 ± 141856.50	0.48
Blood urea nitrogen (mg/dL)	78.60 ± 45.36	84.40 ± 33.65	126.86 ± 37.66	91.70 ± 43.97	<0.01
Baseline serum creatinine (mg/dL)	0.96 ± 0.19	1.09 ± 0.14	1.07 ± 0.24	1.03 ± 0.19	0.05
Peak serum creatinine (mg/dL)	2.11 ± 0.96	2.90 ± 1.95	3.36 ± 1.68	2.66 ± 1.59	<0.01
Serum sodium (mEq/L)	132.85 ± 4.38	132.25 ± 5.27	132.17 ± 4.98	132.49 ± 4.77	0.88
Serum potassium (mEq/L)	3.89 ± 0.74	4.13 ± 0.71	3.98 ± 0.84	3.99 ± 0.75	0.35
Urine sodium (mEq/L)	32.20 ± 26.17	39.22 ± 24.89	52.46 ± 23.87	39.25 ± 26.07	0.01
Fractional excretion of sodium (%)	1.83 ± 2.18	4.04 ± 3.94	4.94 ± 3.49	3.29 ± 3.40	<0.01

## Discussion

This prospective observational study evaluated the prevalence, aetiology, clinical profile, and in-hospital outcomes of AKI among adult patients admitted to a tertiary care centre in South India. Using KDIGO 2012 criteria, AKI was identified in 65 of 2,427 adult General Medicine admissions, corresponding to a prevalence of 2.67% within the study population. This estimate should be interpreted in the context of the study setting, as paediatric, obstetric, surgical, and intensive care admissions were not included. Sepsis was the most common aetiology, accounting for nearly half of all cases, followed by cardiac failure and chronic liver disease. Pre-renal AKI was the predominant subtype and was associated with favourable outcomes, whereas intrinsic renal AKI was more frequently associated with mortality. Complete renal recovery occurred in 43.1% of patients, partial recovery in 33.8%, and in-hospital mortality was observed in 23.1% of cases. Oliguria, hypotension, elevated blood urea nitrogen, peak serum creatinine, urine sodium concentration, FENa, and leukocytosis were significantly associated with poor outcomes.

Comparison with existing literature

The predominance of sepsis as the leading cause of AKI in the present study is consistent with findings from both regional and international literature. Kaaviya et al. [9] reported that infectious diseases constituted the largest contributor to community-acquired AKI in Southern India, while Minja et al. [10] and Hasan et al. [11] identified sepsis and septic shock as major contributors to AKI among critically ill patients. Similarly, Peerapornratana et al. [7] highlighted sepsis-associated AKI as one of the most common forms of AKI worldwide, particularly in low- and middle-income countries where infectious diseases remain a major cause of hospitalisation. The recent Acute Disease Quality Initiative (ADQI) consensus report also recognised sepsis as a leading precipitant of AKI and emphasised its association with adverse clinical outcomes. The high burden of sepsis-associated AKI observed in the present study therefore reflects both regional disease patterns and broader global trends.

The mortality rate observed in our study (23.1%) is comparable to those reported in previous observational studies of AKI. Kaaviya et al. [9] documented a mortality rate of 15.1% among patients with community-acquired AKI, whereas Mahbub et al. [12] reported substantial in-hospital mortality among hospitalised AKI patients. International studies have consistently demonstrated that AKI is associated with increased mortality, particularly when accompanied by sepsis, haemodynamic instability, or multiorgan dysfunction [3,8]. Differences in mortality rates across studies likely reflect variations in patient populations, severity of illness, healthcare resources, and thresholds for intensive care and renal replacement therapy. Nevertheless, the mortality observed in the present study underscores the continued clinical significance of AKI as a major contributor to adverse hospital outcomes.

Our finding that pre-renal AKI was the most common subtype and was associated with more favourable outcomes is consistent with previous literature demonstrating that reversible haemodynamic causes generally carry a better prognosis than established parenchymal injury. Studies by Sujatha and Ramprasad [13] and Tresa et al. [14] reported poorer outcomes among patients with intrinsic renal injury, particularly those with acute tubular necrosis and other forms of structural renal damage. The better outcomes observed among patients with pre-renal AKI in the present study may therefore be explained by the potentially reversible nature of haemodynamic disturbances when recognised and treated promptly. Similarly, Kaaviya et al. [9] and Khan et al. [15] observed that hypovolaemia, infection, and circulatory failure accounted for a substantial proportion of AKI cases in South Asian populations, supporting our finding that pre-renal causes remain a dominant contributor to AKI in this region.

Oliguria and hypotension were significantly associated with adverse outcomes in the present study. Similar observations have been reported by Kaaviya et al. [9], who identified hypotension and reduced urine output as important markers of poor prognosis among patients with AKI. Persistent hypotension can lead to prolonged renal hypoperfusion, worsening tubular injury, and progression to multiorgan dysfunction. Likewise, oliguria is widely recognised as an early clinical marker of severe renal impairment and has been incorporated into the KDIGO diagnostic criteria because of its prognostic significance [1].

Several laboratory parameters, including elevated leukocyte count, blood urea nitrogen, peak serum creatinine, urine sodium concentration, and FENa, were also associated with adverse outcomes. Minja et al. [10] reported similar associations between elevated inflammatory markers, worsening renal dysfunction, and poor clinical outcomes among critically ill patients. Elevated leukocyte counts likely reflect the systemic inflammatory response associated with severe infection and sepsis, while increased blood urea nitrogen and serum creatinine indicate greater impairment of renal function. Higher urine sodium concentrations and FENa may reflect established tubular dysfunction, which is more commonly encountered in intrinsic renal injury and is therefore associated with poorer recovery.

The prevalence of AKI observed in the present study (2.67%) was lower than that reported in intensive care unit-based investigations. Minja et al. [10] reported an AKI prevalence of 55.3% among ICU admissions, while Hasan et al. [11] observed AKI in more than 80% of critically ill patients. This difference is expected because the present study evaluated adult patients admitted to General Medicine wards rather than exclusively critically ill populations. Furthermore, the prevalence reported herein reflects only adult General Medicine admissions and excludes paediatric, obstetric, surgical, and intensive care populations. Variations in study setting, case mix, disease severity, and diagnostic practices are therefore important considerations when comparing epidemiological estimates across studies.

Clinical implications

The present study provides contemporary data on the epidemiology and outcomes of AKI among adult General Medicine admissions in a tertiary care centre in South India. Sepsis accounted for nearly half of all AKI cases, highlighting its continued importance as the predominant precipitating factor in this setting. The study further demonstrated that pre-renal AKI was the most common subtype and was associated with substantially better clinical outcomes than intrinsic renal AKI, suggesting that early identification and correction of reversible haemodynamic abnormalities may improve renal recovery. An important finding of the present study was the significant association of hypotension, oliguria, leukocytosis, elevated blood urea nitrogen, higher peak serum creatinine, urine sodium concentration, and FENa with adverse in-hospital outcomes. These readily available clinical and laboratory parameters may assist clinicians in identifying patients at increased risk of poor outcomes and prioritising closer monitoring and timely intervention. Furthermore, the study provides locally relevant evidence from a South Indian tertiary care setting, where epidemiological patterns and healthcare resources may differ from those reported in high-income countries. Such data may help guide risk stratification strategies and resource allocation in similar healthcare environments.

Strengths and limitations

The strengths of this study include its prospective design, systematic collection of clinical and laboratory data, and use of internationally accepted KDIGO diagnostic criteria, thereby enhancing internal validity and comparability with contemporary literature. Furthermore, the comprehensive assessment of demographic characteristics, clinical presentation, laboratory parameters, aetiology, and in-hospital outcomes provides a detailed overview of AKI among adult patients admitted to a tertiary care setting. Several limitations should be acknowledged. First, this was a single-centre study with a relatively modest sample size, which may limit the generalisability of the findings. Second, only in-hospital outcomes were evaluated, and no post-discharge follow-up was performed to assess long-term renal recovery, progression to chronic kidney disease, or delayed mortality. Third, surgical, obstetric, paediatric, and intensive care unit populations were not included, limiting the applicability of the findings to these patient groups. Consequently, the reported prevalence reflects only adult General Medicine admissions and should not be extrapolated to the overall hospital population. Finally, multivariable regression analysis was not performed because of the limited sample size; therefore, the variables identified in this study should be interpreted as factors associated with adverse outcomes rather than independent predictors of mortality.

## Conclusions

AKI remains an important cause of morbidity and mortality among hospitalised adults in tertiary care centres. Sepsis was the leading aetiology in the present study, while pre-renal AKI represented the most common subtype. Oliguria, hypotension, leukocytosis, elevated blood urea nitrogen, and higher peak serum creatinine were associated with adverse outcomes. These findings highlight the importance of early recognition, prompt treatment of sepsis, optimisation of haemodynamic status, and careful monitoring of high-risk patients. Larger multicentre studies with long-term follow-up are warranted to further define the epidemiology and prognostic factors of AKI in the Indian population.
